# Parkinson's disease candidate gene prioritization based on expression profile of midbrain dopaminergic neurons

**DOI:** 10.1186/1423-0127-17-66

**Published:** 2010-08-17

**Authors:** Shahrooz Vahedi, Mehrnoosh Rajabian, Arman Misaghian, Daniel Grbec, Horst H Simon, Kambiz N Alavian

**Affiliations:** 1 The Bahá'í Institute for Higher Education (BIHE), Tehran, Iran; 2 Interdisciplinary Center for Neuroscience, Ruprecht Karls Universität, Heidelberg, Germany; 3 Department of Internal Medicine, Endocrinology, Yale University, New Haven, CT, USA

## Abstract

**Background:**

Parkinson's disease is the second most common neurodegenerative disorder. The pathological hallmark of the disease is degeneration of midbrain dopaminergic neurons. Genetic association studies have linked 13 human chromosomal loci to Parkinson's disease. Identification of gene(s), as part of the etiology of Parkinson's disease, within the large number of genes residing in these loci can be achieved through several approaches, including screening methods, and considering appropriate criteria. Since several of the indentified Parkinson's disease genes are expressed in substantia nigra pars compact of the midbrain, expression within the neurons of this area could be a suitable criterion to limit the number of candidates and identify PD genes.

**Methods:**

In this work we have used the combination of findings from six rodent transcriptome analysis studies on the gene expression profile of midbrain dopaminergic neurons and the PARK loci in OMIM (Online Mendelian Inheritance in Man) database, to identify new candidate genes for Parkinson's disease.

**Results:**

Merging the two datasets, we identified 20 genes within PARK loci, 7 of which are located in an orphan Parkinson's disease locus and one, which had been identified as a disease gene. In addition to identifying a set of candidates for further genetic association studies, these results show that the criteria of expression in midbrain dopaminergic neurons may be used to narrow down the number of genes in PARK loci for such studies.

## Background

Selective degeneration of mesencephalic dopaminergic (mesDA) neurons of substantia nigra pars compacta (SNpc) is the pathological hallmark of Parkinson's disease (PD; OMIM #168600). Although the molecular mechanism behind demise of these neurons during the course of PD is still unknown, numerous studies have shown contribution of both genetic and environmental and factors, such as neurotoxins, to degeneration of this inherently vulnerable neuronal population [1], with less than 15% of all PD cases account for familial subtype.

Based on DNA linkage studies, 13 distinct human chromosomal locations, PARK loci, have been linked to the disease: PARK1 [2], PARK2 [3], PARK3 [4], PARK4 [5], PARK5 [6], PARK6 [7], PARK7 [8], PARK8 [9], PARK9 [10], PARK10 [11], PARK11 [12], PARK12[12], PARK13 [13]. These loci expand variably, from 7 to 40 Mb, on different chromosomes each of which contains several hundreds of genes. There are 4 orphan PD loci with no associated genes so far and mutation/s in 8 genes, located in 9 out of 13 PARK loci have been linked to PD. Mutations in *α-synuclein *located on PARK1 and PARK 4, *Parkin*, *Ubiquitin carboxy-terminal-hydrolase-L1(UCHL1), PTEN-induced-putative kinase (PINK1), DJ1, Leucine-rich repeat kinase 2 (LRRK2), ATPase type 13A2 (ATP13A2), HTRA2 genes *in PARK2, 5, 6, 7, 8, 9 and 13 have been shown, respectively, to cause PD [14]. Additionally, two other susceptibility genes, *Nurr1 *(NR4A2) and *tau*, which show no linkage to previously described PARK Loci, have been linked to families with Parkinson's disease [15]. Each of the PARK loci contains a large number of genes and identification of disease genes requires proper criteria to narrow down the number of candidates. Since five out of seven PD genes (*α-SYNUCLEIN*, *PARKIN*, *UCHL1, PINK1 *and *LRRK2*) plus the two latter genes (NURR1 and TAU) are expressed in midbrain dopaminergic neurons, possibly linking the abnormality in their expression or structure to selective degeneration of SNpc neurons, expression of genes within this neuronal population seems to be a suitable criterion for narrowing down the number of genes to be further analyzed for identification of PD genes to be associated with orphan PD loci.

To date, six genome-wide screens have been performed to identify gene expression pattern of rodent dopaminergic neurons [16-21]. In a recent study, using the in situ hybridization database, Allen Brain Atlas (ABA) [22], and the results of the transcriptome analyses, we verified the expression of 362 genes within the dopaminergic neurons of the midbrain [23]. In this study, using the criteria of specific expression, and the strategy described by Gherbassi et al. [15], we merged the rodent gene expression data from three of the six screens, with human linkage studies to narrow down the number of candidates for disease susceptibility genes.

## Methods

### Transcript collection and processing

The transcripts of the mouse and rat genes were obtained from six published libraries [16-21]. After elimination of redundancies and duplications, above background mRNA expression within ventral midbrain, with expression patterns, resembling that of tyrosine hydroxylase in VTA or SNpc, was verified in ABA [22], as previously described [23]. We employed each nucleotide sequence for a nucleotide-nucleotide BLAST (blastn) (basic local alignment search tool) on the non-redundant database http://www.ncbi.nlm.nih.gov/BLAST/ and on the mouse genome http://www.ncbi.nlm.nih.gov/genome/seq/BlastGen/BlastGen.cgi?taxid=10090. Using the criteria of highest homology and lowest e-value, for this study, we employed only the unambiguous hits (transcripts), with homology on mouse genome.

### Mapping gene locations into the PARK loci

The human analogs for the mouse genes were found, using the Gene, Protein or the BLAST search functions of the NCBI database. After determining the Genbank accession number of the genes, the cytogenetic location on the human genome was determined using the Map Viewer search tool http://www.ncbi.nlm.nih.gov/mapview/. The neighboring genes on the mouse and human genome were considered to verify the identity and the position of the gene in the human genome. The cytogenetic positions on the human genome were compared with previously described PARK loci http://www.pdgene.org. We aligned the human chromosome map view with the OMIM morbid/disease map http://www.ncbi.nlm.nih.gov/entrez/query.fcgi?db=OMIM to identify the PD gene candidates.

## Results

Majority of the genes associated with familial Parkinson's disease are expressed within mesDA neurons, the population of neurons which is lost during the course of the disease. We, therefore, used the criterion of expression to prioritize the identification of genes, which may be the risk factors or responsible for the onset or progression of the disease. Recently, we identified the expression pattern of the genes within two major nuclei of the midbrain, substantia nigra pars compacta and ventral tegmental area, detectable by in situ hybridization. This was done by verifying the expression pattern of genes from six published libraries [16-21] in the Allen Brain Atlas in situ hybridization database [22] and comparing them to the expression pattern of tyrosine hydroxylase within the midbrain. This search confirmed the expression of 362 genes out of the published libraries within mesDA neurons [23]. The results were confirmed and updated as of May 2010.

### Cytogenetic locations and linkage to human genome

A recent study by Gherbassi et al. considered the genes in three of the six screens (Thuret et al., Stewart et al., and Barrett et al.), which were performed to identify the expression profile of mesDA neurons. In order to avoid redundancies, we excluded the genes, which were identified by more than one screen and only considered the genes identified by Greene et al., Grimm et al., and Chung et al. After confirming the expression of each gene within the mouse ventral midbrain and determining the homologous human gene name, symbol and accession number (Additional file 1: Table S1), we used the Map Viewer search tool http://www.ncbi.nlm.nih.gov/mapview/ on the NCBI web site to determine the cytogenetic locations of 199 genes. Then, the positions were compared with the positions of the PARK loci by aligning the human chromosome map view with the OMIM morbid map in http://www.ncbi.nlm.nih.gov/entrez/query.fcgi?db=OMIM, using the OMIM identifiers 163890, 602544, 602404, 605543, 191342, 605909, 602533, 607060, 606693, 606852, 607688, 300557 and 610297 for PARK1-13. Of 199 genes, the cytogenetic locations of 20 overlapped with that of PARK loci, which were labeled PD candidate genes. One of these genes, UCHL1, is a known PD gene, and 7 were within Park12, which is an orphan PD locus (Table 1).

**Table 1:** PD candidate genes.

	Gene Name	**Accession No**.	Symbol	Screen	Position	Locus
1	Tetraspanin 6	NM_003270.2	Tspan6	Chung et al.	Xq22.1	PARK 12

2	ubiquitin-conjugating enzyme E2K (UBC1 homolog, yeast)	NM_001111112.1	UBE2K	Chung et al.	4p14	PARK 5

3	Ribosomal protein L11	NM_000975.2	RPL11	Chung et al.	1p36.1-p35	PARK 6 & 7

4	Ribosomal protein L36a	NM_021029.4	RPL36A	Chung et al.	Xq22.1	PARK 12

5	SWI/SNF related, matrix associated, actin dependent regulator of chromatin, subfamily a, member 1	NM_003069.3	SMARCA1	Chung et al.	Xq25	PARK 12

6	MARCKS-like 1	NM_023009.5	MARCKSL1	Chung et al.	1p35.1	PARK 6

7	GTPase activating protein 24	NM_001025616.2	ARHGAP24	Chung et al.	4q21.23-4q21.3	PARK 4

8	F-box only protein 2	NM_012168.4	FBXO2	Chung et al.	1p36.22	PARK 7

9	T-complex-associated-testis-expressed 1-like	NM_006520.2	DYNLT3	Chung et al.	Xp21	PARK 12

10	Cyclin I	NM_006835.2	CCNI	Chung et al.	4q21.1	PARK 4

11	Solute carrier family 25 (mitochondrial carrier, adenine nucleotide translocator), member 5	NM_001152.4	SLC25A5	Chung et al.	Xq24-q26	PARK 12

12	Pyruvate dehydrogenase E1 alpha 1	NM_000284.2	PDHA1	Greene et al.	Xp22.12	PARK 12

13	Ubiquitin carboxy-terminal hydrolase L1 PARK	NM_004181.4	UCHL1	Greene et al.	4p14	PARK 5

14	G protein-coupled receptor, family C, group 5, member A	NM_003979.3	GPRC5A	Grimm et al.	12p13.1	PARK 8

15	Protein tyrosin phosphatase, receptor type, U	NM_005704.3	PTPRU	Grimm et al.	1p35.3	PARK 6

16	Klech-like 13 (drosophila)	NM_001168299.1	KLHL13	Grimm et al.	Xq24	PARK 12

17	Dehyrogenase/reductase (SDR family) member 3	NM_004753.4	DHRS3	Grimm et al.	1p36.22-1p36.21	PARK 7

18	NEL-like 2 (chicken)	NM_001145107.1	NELL2	Grimm et al.	12q13.11-q13.12	PARK 8

19	Serin (or cysteine) peptidase inhibitor clade B, member 6a	NM_004568.4	SERPINB6	Grimm et al.	6q25	PARK 2

20	Peripherin 1	NM_006262.3	PRPH	Grimm et al.	12q12-q13	PARK 8

The list of genes, with ISH-detectable expression in midbrain dopaminergic neurons, with cytogenetic locations, falling within PARK loci are shown. Numbers 1,4,5,9,11,12 and 16 are within PARK-12, an orphan PD locus and UCHL1 (13) is a known PD gene.

## Discussion

Identifying multi-factorial disease-related genes requires methods based on priori knowledge about the candidates. To prioritize the genes, several context-based approaches, ranging from phylogenetic profiling to biochemical data integration have been used. Any given method has its advantages and limitations and the ultimate test for validity of each method is functional relevance of the identified candidates to initiation or progression of the disease. In Parkinson's disease the functional validation has been strongly linked to the neuronal population that is afflicted during the course of the disease, the dopaminergic neurons of substantia nigra pars compacta in the midbrain.

Despite being the most prominent pathological feature of PD, the reasons underlying specific degeneration of these neurons are not fully understood. However, identification of disease genes has been crucial in understanding multiple cellular and molecular mechanisms, contributing to this process. The expression of several of the familial PD genes within nesDA neurons seems to be required for physiological functions and survival of this neuronal population. A number of studies have established the role of multiple PD genes, including PARKIN, PINK1 and DJ1 in degradation of unfolded proteins [24]. Several other studies also have established their neuroprotective role within mesDA neurons against mitochondrial dysfunction in the animal models of the disease [25]. PD genes also play vital functions within the dopaminergic synapses [26].

Given these data, it is highly likely that familial Parkinson's disease, caused by the loss of function mutations in the PD genes, is due to hindrance to functions of the wild-type forms and that the proper expression of the genes within mesDA neurons is essential to their long-term survival. Considering this hypothesis, genomic convergence, which combines gene expression with genomic linkage analysis, has been used to prioritize candidate susceptibility genes for PD [15,27]. In this study, we used this approach to find candidates, among the genes that are expressed in mesDA neurons, shown by six rodent studies and verified by using the Allen Brain Atlas in situ hybridization database. A previous study, by Gherbassi et al. had merged the data from three of the six screens to Parkinson's disease linkage studies. Here we found that 20, in addition to 21 human genes, identified by Gherbassi et al., are located in multiple PARK loci (Table 1). The presence of UCHL1, a known PD gene, among the results of this study validates the genomic convergence approach as an efficient tool for prioritization and identification of candidate PD genes. Using the same approach, three other known PD genes (so far, 10% or 4 out of 41 genes, found by two studies, are known PD genes) were identified previously [15]. Additional genetic and disease model studies are needed to determine whether any of the seven genes, which are within PARK12 can be considered a disease gene and their degree of functional relevance to survival and maintenance of mesDA neurons.

## List of abbreviations

PD: Parkinson's disease; mesDA: mesencephalic dopaminergic; OMIM: Online Mendelian Inheritance in Man; BLAST: Basic Local Alignment Search Tool; ISH: in situ hybridization; ABA: Allen Brain Atlas.

## Competing interests

The authors declare that they have no competing interests.

## Authors' contributions

This study was designed and supervised by KNA and was performed by SV, MR. The data was analyzed by KNA and HHS. DG and AM partook in design of the study and made critical comments. SV and KNA drafted the manuscript and all authors read and approved the final version.

## Supplementary Material

Additional file 1 **Table S1: The list of genes considered for prioritization in this study**. The genes showing above background expression levels in midbrain dopaminergic neurons, confirmed in using ABA in situ hybridization database, after the removal of redundancies with previous studies. Click here for file
