# Supplementary material for: Parkinson's disease candidate gene prioritization based on expression profile of midbrain dopaminergic neurons
Source: J Biomed Sci. 2010 Aug 17;17(1):66. doi: 10.1186/1423-0127-17-66 (PMC2929225; doi:10.1186/1423-0127-17-66)
Supplement: Additional file 1 — Table S1: The list of genes considered for prioritization in this study. The genes showing above background expression levels in midbrain dopaminergic neurons, confirmed in using ABA in situ hybridization database, after the removal of redundancies with previous studies. [file 1423-0127-17-66-S1.PDF]

	Gene Name	Gene Symbol	Accession Number	Cytogenic Location
1	aldo-keto reductase family 1, member B1 (aldose reductase)	AKR1B1	NM_001628.2	7q35
2	cholinergic receptor, nicotinic, alpha 4	CHRNA4	NM_000744.5	20q13.33
3	GDNF family receptor alpha 1	GFRA1	NM_001145453.1	10q25.3
4	tyrosine hydroxylase	TH	NM_000360.3	11p15.5
5	peripherin	PRPH	NM_006262.3	12q12-q13
6	dopa decarboxylase (aromatic L-amino acid decarboxylase)	DDC	NM_000790.3	7p12.2
7	pterin-4 alpha-carbinolamine dehydratase/dimerization cofactor of hepatocyte nuclear factor 1 alpha	PCBD1	NM_000281.2	10q22
8	cell growth regulator with EF-hand domain 1	CGREF1	NM_001166239.1	2p23.3
9	calcyon neuron-specific vesicular protein	CALY	NM_015722.3	10q26.3
10	protein arginine methyltransferase 2	PRMT2	NM_001535.2	21q22.3
11	TSPY-like 2	TSPYL2	NM_022117.3	Xp11.22
12	aldehyde dehydrogenase 1 family, member A1	ALDH1A1	NM_000689.3	9q21.13
13	regulator of calcineurin 2	RCAN2	NM_005822.2	6q12.3
14	RNA binding motif, single stranded interacting protein	RBMS3	NM_001003792.1	3p24.1
15	protein kinase C, alpha	PRKCA	NM_002737.2	17q24.1-17q24.2
16	LY6/PLAUR domain containing 1	LYPD1	NM_001077427.2	2q21.2
17	solute carrier family 39 (zinc transporter), member 4	SLC39A4	NM_017767.2	8q24.3
18	serpin peptidase inhibitor, clade B (ovalbumin), member 6	SERPINB6	NM_004568.4	6q25
19	NEL-like 2 (chicken)	NELL2	NM_001145107.1	12q13.11-q13.12
20	potassium voltage-gated channel, Shal-related subfamily, member 3	KCND3	NM_004980.3	1p13.3
21	protein kinase C, epsilon	PRKCE	NM_005400.2	2p21
22	leucine rich repeat containing 3B	LRRC3B	NM_052953.2	3p24.1
23	serine threonine kinase 39 (STE20/SPS1 homolog, yeast)	STK39	NM_013233.2	2q24.3
24	glucan (1,4-alpha-), branching enzyme 1	GBE1	NM_000158.3	3p12.3-3p12.2
25	dehydrogenase/reductase (SDR family) member 3	DHRS3	NM_004753.4	1p36.22-1p36.21
26	kelch-like 13 (Drosophila)	KLHL13	NM_001168299.1	Xq24
27	aurora kinase A	AURKA	NM_003600.2	20q13.2-q13.3
28	LIM domain only 4	LMO4	NM_006769.3	1p22.3
29	phospholipase C, beta 1 (phosphoinositide-specific)	PLCB1	NM_015192.2	20p12.3-20p12.2
30	cytochrome b-561	CYB561	NM_001017916.1	17q23.3
31	CUG triplet repeat, RNA binding protein 2	CUGBP2	NM_001025076.2	10p14
32	latrophilin 2	LPHN2	NM_012302.2	1p31.1
33	protein tyrosine phosphatase, receptor type, U	PTPRU	NM_005704.3	1p35.3
34	solute carrier organic anion transporter family, member 4A1	SLCO4A1	NM_016354.3	20q13.33
35	cadherin 13, H-cadherin (heart)	CDH13	NM_001257.3	16q23.2-16q24.1
36	serpin peptidase inhibitor, clade E (nexin, plasminogen activator inhibitor type 1), member 2	SERPINE2	NM_001136528.1	2q33-q35
37	pleckstrin homology domain containing, family A member 7	PLEKHA7	NM_175058.4	11p15.1
38	angiomotin like 1	AMOTL1	NM_130847.2	11q14.3
39	pre-B-cell leukemia homeobox 1	PBX1	NM_002585.2	1q23
40	ets variant 5	ETV5	NM_004454.2	3q27.2
41	Purkinje cell protein 4 like 1	PCP4L1	NM_001102566.1	1q23.3

42	lysophosphatidic acid receptor 1	LPAR1	NM_001401.3	9q31.3
43	teashirt zinc finger homeobox 1	TSHZ1	NM_005786.4	18q22.3
44	glutamate receptor interacting protein 2	GRIP2	NM_001080423.1	3p25.1
45	par-3 partitioning defective 3 homolog (C. elegans)	PARD3	NM_019619.2	10p11.22-10p11.21
46	Ras association (RalGDS/AF-6) domain family member 6	RASSF6	NM_177532.3	4q13.3
47	eyes absent homolog 2 (Drosophila)	EYA2	NM_005244.4	20q13.12
48	zinc finger homeobox 3	ZFHX3	NM_001164766.1	16q22.3-q23.1
49	solute carrier family 31 (copper transporters), member 1	SLC31A1	NM_001859.3	9q32
50	glycoprotein (transmembrane) nmb	GPNMB	NM_001005340.1	7q15
51	Ca++-dependent secretion activator 2	CADPS2	NM_001009571.3	7q31.3
52	fatty acid desaturase 3	FADS3	NM_021727.3	11q12.2-11q12.3
53	calcium-sensing receptor	CASR	NM_000388.3	3q13.33-3q21.1
54	transmembrane inner ear	TMIE	NM_147196.2	3p21.31
55	glutathione peroxidase 3 (plasma)	GPX3	NM_002084.3	5q23
56	fibronectin type III domain containing 3B	FNDC3B	NM_001135095.1	3q26.31
57	protein phosphatase 1, regulatory (inhibitor) subunit 14B	PPP1R14B	NM_138689.2	11q13
58	myosin VB	MYO5B	NM_001080467.2	18q21.1
59	phosphofructokinase, liver	PFKL	NM_002626.4	21q22.3
60	NK2 homeobox 1	NKX2-1	NM_001079668.2	14q13.3
61	claudin 1	CLDN1	NM_021101.3	3q28-q29
62	guanine nucleotide binding protein (G protein), gamma 11	GNG11	NM_004126.3	7q21.3
63	stimulator of chondrogenesis 1	SCRG1	NM_007281.2	4q34.1
64	X-box binding protein 1	XBP1	NM_001079539.1	22q12.1
65	G protein-coupled receptor, family C, group 5, member A	GPRC5A	NM_003979.3	12p13.1
66	phosphoglucomutase 2-like 1	PGM2L1	NM_173582.3	11q13.4
67	regulator of G-protein signaling 2, 24kDa	RGS2	NM_002923.3	1q31
68	cytochrome P450, family 51, subfamily A, polypeptide 1	CYP51A1	NM_000786.3	7q21.2-q21.3
69	ubiquitin carboxyl-terminal esterase L1 (ubiquitin thiolesterase)	UCHL1	NM_004181.4	4p14
70	brain-derived neurotrophic factor	BDNF	NM_001143805.1	11p13
71	pyruvate dehydrogenase (lipoamide) alpha 1	PDHA1	NM_000284.2	Xp22.12
72	phospholipase C, beta 4	PLCB4	NM_000933.2	20p12.3-20p12.2
73	lysophospholipase I	LYPLA1	NM_006330.2	8q11.23
74	solute carrier family 24 (sodium/potassium/calcium exchanger), member 2	SLC24A2	NM_020344.1	9p22.1-9p21.3
75	ATPase, Ca++ transporting, cardiac muscle, slow twitch 2	ATP2A2	NM_001135765.1	12q24.11
76	calpain, small subunit 1	CAPNS1	NM_001003962.1	19q13.12
77	inositol 1,4,5-triphosphate receptor, type 1	ITPR1	NM_001099952.2	3p26.2
78	cysteine-rich protein 2	CRIP2	NM_001312.2	14q32.3
79	dynein, cytoplasmic 1, intermediate chain 1	DYNC1I1	NM_001135556.1	7q21.3-q22.1
80	ubiquinol-cytochrome c reductase, Rieske iron-sulfur polypeptide 1	UQCRFS1	NM_006003.2	19q12
81	ATP synthase, H+ transporting, mitochondrial F1 complex, alpha subunit 1, cardiac muscle	ATP5A1	NM_001001937.1	18q21.1
82	glutamic-oxaloacetic transaminase 2, mitochondrial (aspartate aminotransferase 2)	GOT2	NM_002080.2	16q21
83	pyruvate kinase, muscle	PKM2	NM_002654.3	15q22

84	creatine kinase, brain	CKB	NM_001823.3	14q32
85	adenylate kinase 1	AK1	NM_000476.2	9q34.1
86	guanine nucleotide binding protein (G protein), alpha activating activity polypeptide O	GNAO1	NM_020988.2	16q13
87	enolase 3 (beta, muscle)	ENO3	NM_001976.3	17p13.2
88	dopamine receptor D2	DRD2	NM_000795.3	11q23
89	calcium channel, voltage-dependent, T type, alpha 1G subunit	CACNA1G	NM_018896.3	17q21.33
90	protein kinase C, beta	PRKCB	NM_002738.6	16p12.1
91	protein kinase, cAMP-dependent, regulatory, type II, beta	PRKAR2B	NM_002736.2	7q22.3
92	proprotein convertase subtilisin/kexin type 2	PCSK2	NM_002594.2	20p12.1
93	neuritin 1	NRN1	NM_016588.2	6p25.1
94	monoamine oxidase A	MAOA	NM_000240.2	Xp11.3
95	GTP cyclohydrolase 1	GCH1	NM_000161.2	14q22.2-14q22.3
96	solute carrier family 7 (cationic amino acid transporter, y+ system), member 3	SLC7A3	NM_001048164.1	Xq13.1
97	cathepsin D	CTSD	NM_001909.3	11p15.5
98	vesicle-associated membrane protein 2 (synaptobrevin 2)	VAMP2	NM_014232.2	17p13.1
99	transducin-like enhancer of split 1 (E(sp1) homolog, Drosophila)	TLE1	NM_005077.3	9q21.31-9q21.32
100	growth factor receptor-bound protein 10	GRB10	NM_001001549.2	7p12.3-7p12.1
101	target of myb1-like 2 (chicken)	TOM1L2	NM_001033551.2	17p11.2
102	calbindin 1, 28kDa	CALB1	NM_004929.2	8q21.3-q22.1
103	GDP dissociation inhibitor 2	GDI2	NM_001115156.1	10p15
104	LAG1 homolog, ceramide synthase 4	LASS4	NM_024552.2	19p13.2
105	microtubule-associated protein 1 light chain 3 alpha	MAP1LC3A	NM_032514.2	20q11.22
106	nuclear receptor interacting protein 3	NRIP3	NM_020645.2	11p15.4
107	CD24 molecule	CD24	NM_013230.2	6q21
108	RAB3C, member RAS oncogene family	RAB3C	NM_138453.2	5q11.2
109	acyl-CoA synthetase long-chain family member 6	ACSL6	NM_001009185.1	5q31.1
110	myosin VA (heavy chain 12, myoxin)	MYO5A	NM_000259.3	15q21.2
111	RAR-related orphan receptor A	RORA	NM_002943.3	15q22.2
112	syndecan 2	SDC2	NM_002998.3	8q22.1
113	fibroblast growth factor 1 (acidic)	FGF1	NM_000800.3	5q31
114	poliovirus receptor-related 3	PVRL3	NM_015480.1	3q13.13
115	glutamate receptor, ionotropic, N-methyl D-aspartate 2C	GRIN2C	NM_000835.3	17q25
116	zinc finger, DHHC-type containing 2	ZDHHC2	NM_016353.4	8p21.3-p22
117	SATB homeobox 1	SATB1	NM_001131010.1	3p24.3
118	acyl-Coenzyme A dehydrogenase, long chain	ACADL	NM_001608.3	2q34-q35
119	solute carrier family 25 (mitochondrial carrier; adenine nucleotide translocator), member 5	SLC25A5	NM_001152.4	Xq24-q26
120	vav 3 guanine nucleotide exchange factor	VAV3	NM_001079874.1	1p13.3
121	calcium channel, voltage-dependent, alpha 2/delta subunit 3	CACNA2D3	NM_018398.2	3p21.1-3p14.3
122	synuclein, gamma (breast cancer-specific protein 1)	SNCG	NM_003087.2	10q23.2-q23.3
123	oxysterol binding protein-like 11	OSBPL11	NM_022776.4	3q21.2
124	Lix1 homolog (chicken)	LIX1	NM_153234.4	5q15
125	annexin A1	ANXA1	NM_000700.1	9q12-q21.2

126	Ca++-dependent secretion activator	CADPS	NM_003716.3	3p14.2
127	RAS-like, family 11, member B	RASL11B	NM_023940.2	4q12
128	polymerase (DNA directed), beta	POLB	NM_002690.1	8p11.21
129	cyclin I	CCNI	NM_006835.2	4q21.1
130	RNA terminal phosphate cyclase domain 1	RTCD1	NM_001130841.1	1p21.2
131	lysosomal protein transmembrane 4 beta	LAPTM4B	NM_018407.4	8q22.1
132	cadherin 8, type 2	CDH8	NM_001796.2	16q21
133	ganglioside-induced differentiation-associated protein 1	GDAP1	NM_001040875.1	8q21.11
134	adenosine kinase	ADK	NM_001123.2	10q22.2
135	dynein, light chain, Tctex-type 3	DYNLT3	NM_006520.2	Xp.21
136	aspartyl aminopeptidase	DNPEP	NM_012100.2	2q35
137	regulator of G-protein signaling 8	RGS8	NM_001102450.1	1q25
138	pre-B-cell leukemia homeobox 3	PBX3	NM_001134778.1	9q33-q34
139	trophoblast glycoprotein	TPBG	NM_001166392.1	6q14.1
140	CDP-diacylglycerol synthase (phosphatidate cytidylyltransferase) 2	CDS2	NM_003818.2	20p13
141	myocyte enhancer factor 2A	MEF2A	NM_001130926.1	15q26.3
142	mitogen-activated protein kinase 9	MAPK9	NM_001135044.1	5q35
143	F-box protein 2	FBXO2	NM_012168.4	1p36.22
144	macrophage migration inhibitory factor (glycosylation-inhibiting factor)	MIF	NM_002415.1	22q11.23
145	NADH dehydrogenase (ubiquinone) Fe-S protein 8, 23kDa (NADH-coenzyme Q reductase)	NDUFS8	NM_002496.2	11q13
146	ELOVL family member 6, elongation of long chain fatty acids (FEN1/Elo2, SUR4/Elo3-like, yeast)	ELOVL6	NM_001130721.1	4q25
147	CD47 molecule	CD47	NM_001025079.1	3q13.12
148	citrate synthase	CS	NM_004077.2	12q13.2-q13.3
149	phosphatidylinositol transfer protein, alpha	PITPNA	NM_006224.3	17p13.3
150	homer homolog 2 (Drosophila)	HOMER2	NM_004839.2	15q25.2
151	RASD family, member 2	RASD2	NM_014310.3	22q12.3
152	thioredoxin-related transmembrane protein 2	TMX2	NM_001144012.1	11q12.1
153	3-oxoacid CoA transferase 1	OXCT1	NM_000436.3	5p13.1
154	translocase of outer mitochondrial membrane 20 homolog (yeast)	TOMM20	NM_014765.2	1q42.3
155	N-ethylmaleimide-sensitive factor attachment protein, beta	NAPB	NM_022080.2	20p12.3-p11.21
156	amyloid beta (A4) precursor protein-binding, family A, member 2	APBA2	NM_001130414.1	15q11-q12
157	NIMA (never in mitosis gene a)-related kinase 7	NEK7	NM_133494.2	1q31.3
158	vacuolar protein sorting 35 homolog (S. cerevisiae)	VPS35	NM_018206.4	16q11.2
159	RAB6A, member RAS oncogene family	RAB6A	NM_002869.4	11q13.3
160	very low density lipoprotein receptor	VLDLR	NM_001018056.1	9p24
161	Rho GTPase activating protein 24	ARHGAP24	NM_001025616.2	4q21.23-4q21.3
162	visinin-like 1	VSNL1	NM_003385.4	2p24.2
163	protein tyrosine phosphatase, non-receptor type 5 (striatum-enriched)	PTPN5	NM_001039970.1	11p15.1
164	prohibitin 2	PHB2	NM_001144831.1	12p13.31
165	NADH dehydrogenase (ubiquinone) Fe-S protein 2, 49kDa (NADH-coenzyme Q reductase)	NDUFS2	NM_001166159.1	1q23
166	transducin-like enhancer of split 3 (E(sp1) homolog, Drosophila)	TLE3	NM_001105192.1	15q22
167	tachykinin receptor 3	TACR3	NM_001059.1	4q24

168	serine peptidase inhibitor, Kunitz type, 2	SPINT2	NM_001166103.1	19q13.2
169	colony stimulating factor 2 receptor, beta, low-affinity (granulocyte-macrophage)	CSF2RB	NM_000395.2	22q13.1
170	reticulocalbin 1, EF-hand calcium binding domain	RCN1	NM_002901.2	11p13
171	collagen, type XI, alpha 1	COL11A1	NM_001168249.1	1p21
172	FXYD domain containing ion transport regulator 6	FXYD6	NM_001164831.1	11q23.3
173	calbindin 2	CALB2	NM_001740.3	16q22.3
174	MARCKS-like 1	MARCKSL1	NM_023009.5	1p35.1
175	cholecystokinin	CCK	NM_000729.3	3p22-p21.3
176	mesoderm development candidate 2	MESDC2	NM_015154.1	15q13
177	huntingtin-associated protein 1	HAP1	NM_001079870.1	17q21.2
178	SWI/SNF related, matrix associated, actin dependent regulator of chromatin, subfamily a, member 1	SMARCA1	NM_003069.3	Xq25
179	dedicator of cytokinesis 6	DOCK6	NM_020812.2	19p13.2
180	CaM kinase-like vesicle-associated	CAMKV	NM_024046.3	3p21.31
181	isocitrate dehydrogenase 1 (NADP+), soluble	IDH1	NM_005896.2	2q33.3
182	nucleobindin 2	NUCB2	NM_005013.2	11p15.1-p14
183	ribosomal protein L36a	RPL36A	NM_021029.4	Xq22.1
184	lipoprotein lipase	LPL	NM_000237.2	8p22
185	adenylate cyclase activating polypeptide 1 (pituitary)	ADCYAP1	NM_001099733.1	18p11.32
186	translocase of inner mitochondrial membrane 10 homolog (yeast)	TIMM10	NM_012456.2	11q12.1
187	asparagine-linked glycosylation 2, alpha-1,3-mannosyltransferase homolog (S. cerevisiae)	ALG2	NM_033087.3	9q22.33
188	calcium channel, voltage-dependent, beta 2 subunit	CACNB2	NM_000724.3	10p12.33
189	carbonic anhydrase IV	CA4	NM_000717.3	17q23
190	dickkopf homolog 3 (Xenopus laevis)	DKK3	NM_001018057.1	11p15.3
191	dystrophia myotonica, WD repeat containing	DMWD	NM_004943.1	19q13.32
192	EPH receptor A6	EPHA6	NM_001080448.2	3q11.2
193	non-metastatic cells 1, protein (NM23A) expressed in	NME1	NM_000269.2	17q21.33
194	glutamate receptor, ionotropic, kainate 3	GRIK3	NM_000831.3	1p34.3
195	heme oxygenase (decycling) 2	HMOX2	NM_001127204.1	16p13.3
196	histone cluster 3, H2a	HIST3H2A	NM_033445.2	1q42.13
197	HIV-1 Tat interactive protein 2, 30kDa	HTATIP2	NM_001098520.1	11p15.1
198	mannosyl (alpha-1,6-)-glycoprotein beta-1,6-N-acetyl-glucosaminyltransferase, isozyme B	MGAT5B	NM_144677.2	17q25.2
199	tetraspanin 6	TSPAN6	NM_003270.2	Xq22.1
